# Recurrent deletions in the SARS-CoV-2 spike glycoprotein drive antibody escape

**DOI:** 10.1126/science.abf6950

**Published:** 2021-02-03

**Authors:** Kevin R. McCarthy, Linda J. Rennick, Sham Nambulli, Lindsey R. Robinson-McCarthy, William G. Bain, Ghady Haidar, W. Paul Duprex

**Affiliations:** 1 Center for Vaccine Research, University of Pittsburgh School of Medicine, Pittsburgh, PA, USA.; 2 Department of Microbiology and Molecular Genetics, University of Pittsburgh School of Medicine, Pittsburgh, PA, USA.; 3 Laboratory of Molecular Medicine, Boston Children’s Hospital, Harvard Medical School, Boston, MA, USA.; 4 Department of Genetics, Harvard Medical School, Boston, MA, USA.; 5 Division of Pulmonary, Allergy, and Critical Care Medicine, Department of Internal Medicine, UPMC, Pittsburgh, PA, USA.; 6 Division of Pulmonary, Allergy, and Critical Care Medicine, Department of Medicine, University of Pittsburgh School of Medicine, Pittsburgh, PA, USA.; 7 VA Pittsburgh Healthcare System, Pittsburgh, PA, USA.; 8 Division of Infectious Disease, Department of Medicine, University of Pittsburgh School of Medicine, Pittsburgh, PA, USA.; 9 Division of Infectious Disease, Department of Internal Medicine, UPMC, Pittsburgh, PA, USA.

## Abstract

Zoonotic pandemics, such as that caused by severe acute respiratory syndrome coronavirus 2 (SARS-CoV-2), can follow the spillover of animal viruses into highly susceptible human populations. The descendants of these viruses have adapted to the human host and evolved to evade immune pressure. Coronaviruses acquire substitutions more slowly than other RNA viruses. In the spike glycoprotein, we found that recurrent deletions overcome this slow substitution rate. Deletion variants arise in diverse genetic and geographic backgrounds, transmit efficiently, and are present in novel lineages, including those of current global concern. They frequently occupy recurrent deletion regions (RDRs), which map to defined antibody epitopes. Deletions in RDRs confer resistance to neutralizing antibodies. By altering stretches of amino acids, deletions appear to accelerate SARS-CoV-2 antigenic evolution and may, more generally, drive adaptive evolution.

## Eluding detection

Influenza viruses evade immunity initiated by previous infection, which explains recurrent influenza pandemics. Unlike the error-prone RNA-dependent RNA polymerase of influenza, severe acute respiratory syndrome coronavirus 2 (SARS-CoV-2) and related viruses contain polymerases with proofreading activity. However, proofreading cannot correct deletions, which during a long-term persistent infection could result in the generation of viruses showing alteration of entire stretches of amino acids and the structures they form. McCarthy *et al.* identified an evolutionary signature defined by prevalent and recurrent deletions in the spike protein of SARS-CoV-2 at four antigenic sites. Deletion variants show human-to-human transmission of viruses with altered antigenicity.

*Science*, this issue p. 1139

## Teaser

SARS-CoV-2 genome deletions that arise independently in persistently infected individuals show recurrent and convergent evolution.

Severe acute respiratory syndrome coronavirus 2 (SARS-CoV-2) emerged from a yet-to-be-defined animal reservoir and initiated a pandemic in 2020 (*1*–*5*). It has acquired limited adaptions, most notably the Asp^614^ → Gly (D614G) substitution in the spike (S) glycoprotein (*6*–*8*). Humoral immunity to S glycoprotein appears to be the strongest correlate of protection (*9*), and recently approved vaccines deliver this antigen by immunization. Coronaviruses such as SARS-CoV-2 acquire substitutions slowly as the result of a proofreading RNA-dependent RNA polymerase (RdRp) (*10*, *11*). Other emerging respiratory viruses have produced pandemics followed by endemic human-to-human spread. The latter is often contingent upon the introduction of antigenic novelty that enables reinfection of previously immune individuals. Whether SARS-CoV-2 S glycoprotein will evolve altered antigenicity, or specifically how it may change in response to immune pressure, remains unknown. We and others have reported the acquisition of deletions in the N-terminal domain (NTD) of the S glycoprotein during long-term infections of immunocompromised patients (*12*–*15*). We have identified this as an evolutionary pattern defined by recurrent deletions that alter defined antibody epitopes. Unlike substitutions, deletions cannot be corrected by proofreading activity, and this may accelerate adaptive evolution in SARS-CoV-2.

**Fig. 1:**
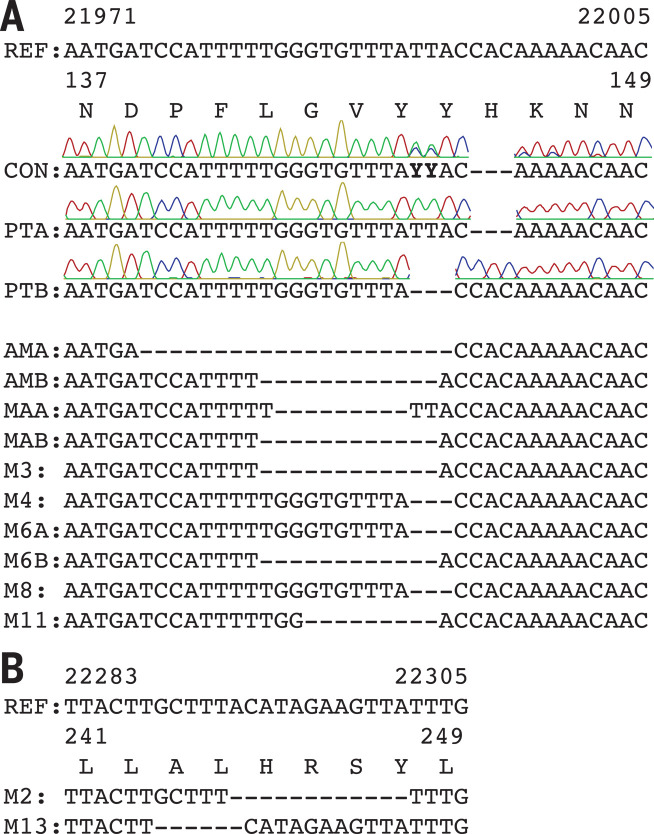
Deletions in SARS-CoV-2 spike glycoprotein arise during persistent infections of immunosuppressed patients. (**A**) Top: Sequences of viruses isolated from PLTI1 (PT) and viruses from patients with deletions in the same NTD region. Chromatograms are shown for sequences from PLTI1, which include sequencing of bulk reverse transcription products (CON) and individual cDNA clones. Bottom: Sequences from other long-term infections from individuals AM (*18*), MA-JL (MA) (*19*), and a MSK cohort (M) with individuals 3, 4, 6, 8, and 11 (*13*). Letters (A and B) designate different variants from the same patient. (**B**) Sequences of viruses from two patients (M2 and M13) with deletions in a different region of the NTD. All sequences are aligned to reference sequence (REF) MN985325 (WA-1). See fig. S1 for genetic analysis of patient isolates. Amino acid abbreviations: A, Ala; D, Asp; F, Phe; G, Gly; H, His; K, Lys; L, Leu; N, Asn; P, Pro; R, Arg; S, Ser; V, Val; Y, Tyr.

An immunocompromised cancer patient infected with SARS-CoV-2 was unable to clear the virus and succumbed to the infection 74 days after COVID-19 diagnosis (*15*). Treatment included remdesivir, dexamethasone, and two infusions of convalescent serum. We designate this individual as Pittsburgh long-term infection 1 (PLTI1). We consensus-sequenced and cloned S genes directly from clinical material obtained 72 days after COVID-19 diagnosis and identified two variants with deletions in the NTD (Fig. 1A).

These data from PLTI1 and a similar report (*12*) prompted us to interrogate patient metadata sequences deposited in GISAID (*16*). In searching for similar viruses, we identified eight patients with deletions in the S glycoproteins of viruses sampled longitudinally over a period of weeks to months (Fig. 1A and fig. S1A). For each, early time points had intact S sequences and later time points had deletions within the S gene. Six had deletions that were identical to, overlapping with, or adjacent to those in PLTI1. Deletions at a second site were present in viruses isolated from two other patients (Fig. 1B); reports on these patients have since been published (*13*, *14*). Viruses from all but one patient could be distinguished from one another by nucleotide differences present at both early and late time points (fig. S1B). On a tree of representative contemporaneously circulating isolates, they form monophyletic clades, making either a second community-acquired or nosocomially acquired infection unlikely (fig. S1C). The most parsimonious explanation is that these deletions arose independently as the result of a common selective pressure to produce strikingly convergent outcomes.

**Fig. 2:**
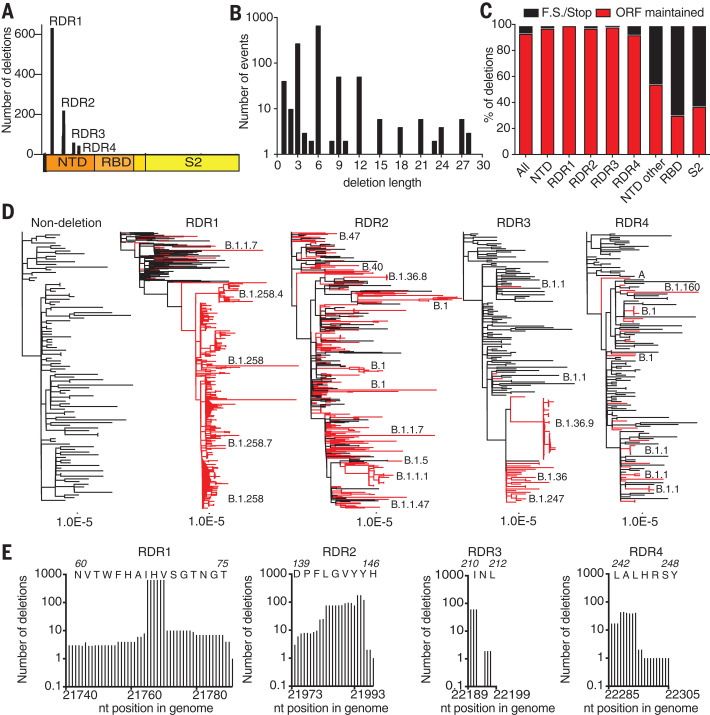
Identification and characterization of recurrent deletion regions in SARS-CoV-2 spike protein. (**A**) Positional quantification of deleted nucleotides in S among GISAID sequences. We designate the four clusters as recurrent deletion regions (RDRs) 1 to 4. (**B**) Length distribution of deletions. (**C**) The percentage of deletion events at the indicated site that either maintain the open reading frame (ORF) or introduce a frameshift or premature stop codon (F.S./Stop). (**D**) Phylogenetic analysis of deletion variants (red branches) and genetically diverse nondeletion variants (black branches). Specific deletion clades/lineages are identified. Maximum likelihood phylogenetic trees, rooted on NC_045512, were calculated with 1000 bootstrap replicates. Trees with branch labels are in fig. S2. (**E**) Abundance of nucleotide (nt) deletions in each RDR. Positions are defined by reference sequence MN985325, by codon (top) and nucleotide (below). Amino acid abbreviations: A, Ala; D, Asp; F, Phe; G, Gly; H, His; I, Ile; L, Leu; N, Asn; P, Pro; R, Arg; S, Ser; T, Thr; V, Val; W, Trp; Y, Tyr.

We searched the GISAID sequence database (*16*) for additional instances of deletions within S glycoproteins. From a dataset of 146,795 sequences (deposited from 1 December 2019 to 24 October 2020) we identified 1108 viruses with deletions in the S gene. When mapped to the S gene, 90% of these deletions occupied four discrete sites within the NTD (Fig. 2A). We term these important sites recurrent deletion regions (RDRs), numbering them 1 to 4 from the 5′ to the 3′ end of the S gene. Deletions identified in patient samples correspond to RDR2 (Fig. 1A) and RDR4 (Fig. 1B). Most deletions appear to have arisen and been retained in replication-competent viruses. Without selective pressure, in-frame deletions should occur one-third of the time. However, we observed a preponderance of in-frame deletions with lengths of 3, 6, 9, and 12 (Fig. 2B). Among all deletions, 93% are in frame and do not produce a stop codon (Fig. 2C). In the NTD, >97% of deletions maintain the open reading frame. Other S glycoprotein domains do not follow this trend; for example, deletions in the receptor binding domain (RBD) and S2 preserve the reading frame 30% and 37% of the time, respectively.

To trace the origins of RDR variants, we produced phylogenies for each with 101 additional genomes that sample much of the genetic diversity within the pandemic (Fig. 2D). The RDR variants interleave with nondeletion sequences and occupy distinct branches, indicating their recurrent generation. This is most pronounced for RDRs 1, 2, and 4 but is also true of RDR3, with conservatively four independent instances. RDR variants form distinct lineages/branches, most prominently in RDR1 (lineage B.1.258), and suggest human-to-human transmission events. Using sequences with sufficient metadata to explicitly differentiate individuals, we verified the transmission of a variant within each RDR between people (fig. S2).

We defined the RDRs on the basis of peaks in the spectrum of S glycoprotein deletions. Deletion lengths and positions vary within RDRs 1, 2, and 4 (Fig. 2E). Variation is greatest in RDRs 2 and 4, with the loss of S glycoprotein residues 144/145 (adjacent tyrosine codons) in RDR2 and residues 243 and 244 in RDR4 appearing to be favored. In contrast, the loss of residues 69 and 70 accounts for the vast majority of RDR1 deletions. On the basis of our phylogenetic analysis and accompanying lineage classifications, this two–amino acid deletion has arisen independently at least 13 times. RDR3 largely consists of three nucleotide deletions in codon 220.

**Fig. 3:**
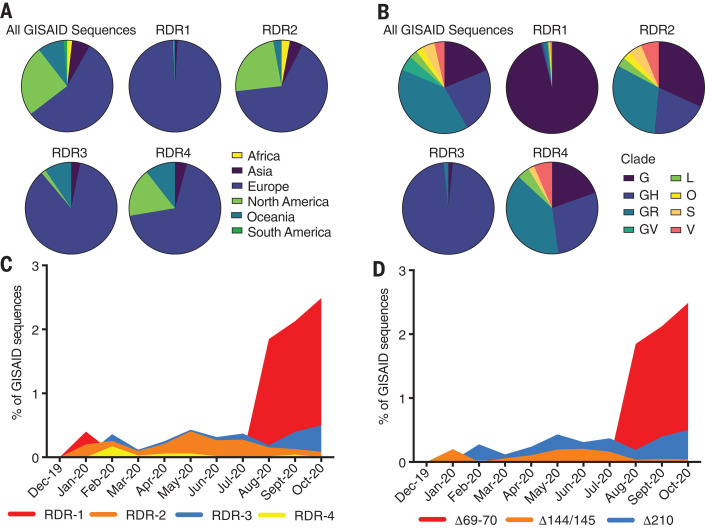
Geographic, genetic, and temporal abundance of RDR variants. (**A** and **B**) Geographic (A) and genetic (B) distributions of RDR variants compared to the GISAID database (sequences from 1 December 2019 to 24 October 2020). GISAID clade classifications are used in (B). (**C**) Frequency of RDR variants among all complete genomes deposited in GISAID. (**D**) Frequency of specific RDR deletion variants (numbered according to spike amino acids) among all GISAID variants. The plot of RDR3/Δ210 has been adjusted by 0.02 units on the *y* axis for visualization in (C) because of its overlap with RDR2, and this adjustment has been retained in (D) to enable direct comparisons between panels.

We evaluated the genetic, geographic, and temporal sampling of RDR variants (Fig. 3, A and B). This analysis was limited to sequences deposited in GISAID (*16*) where sequences from specific nations and regions are overrepresented (e.g., United Kingdom and other European countries). We show the distribution of all sequences within the database for reference. For RDR2 and RDR4, the genetic and geographic distributions largely mirror those of reported sequences. Variants of RDR1 and RDR3 are strongly polarized to specific clades and geographies. This is likely the result of successful lineages circulating in regions with strong sequencing initiatives. Our temporal analysis indicates that RDR variants have been present throughout the pandemic (Fig. 3C). Specific variant lineages such as B.1.258 (Fig. 2D) harboring Δ69–70 in RDR1 have rapidly risen to notable abundance (Fig. 3D). Circulation of B.1.36 with RDR3 Δ210 accounts for most of the RDR3 examples (Fig. 2D and Fig. 3, C and D). The abundance of RDR2 Δ144/145 is explained by independent deletion events followed by transmission (Fig. 2D and Fig. 3, C and D).

**Fig. 4:**
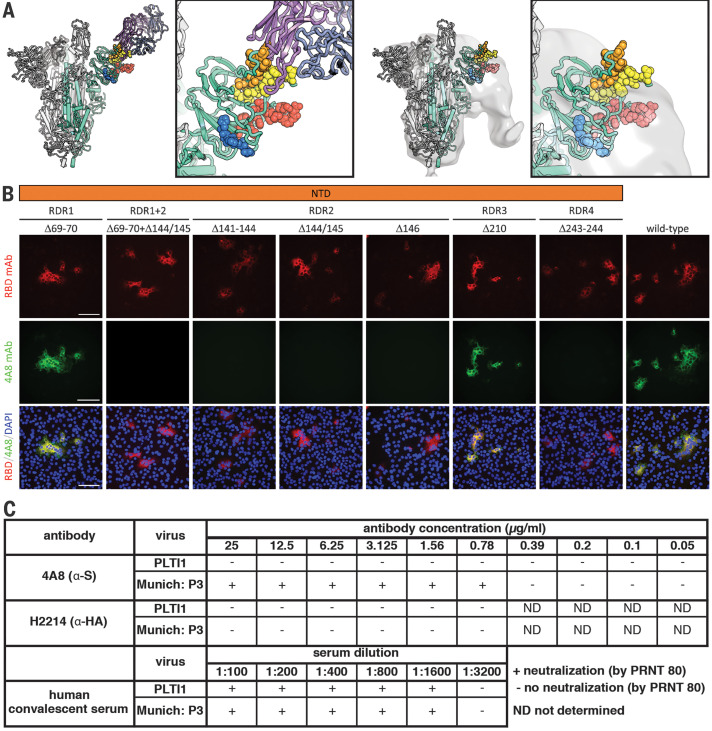
Deletions in the spike NTD alter its antigenicity; RDRs map to defined antigenic sites. (**A**) Left: A structure of antibody 4A8 (*17*) (PDB ID 7C21) (purple) bound to one protomer (green) of a SARS-CoV-2 spike trimer (gray). RDRs 1 to 4 are colored red, orange, blue, and yellow, respectively, and are shown as spheres. The boxed image is a close-up of the interaction site. Right: The electron microscopy density of COV57 serum Fabs (*18*) (EMDB emd_22125) fit to SARS-CoV-2 S glycoprotein trimer (PDB ID 7C21). The boxed image is a close-up of the interaction site. (**B**) S glycoprotein distribution in Vero E6 cells at 24 hours after transfection with S protein deletion mutants, visualized by indirect immunofluorescence in permeabilized cells. A monoclonal antibody to SARS-CoV-2 S protein receptor-binding domain (RBD mAb; red) detects all mutant forms of the protein (Δ69–70, Δ69–70+Δ141–144, Δ141–144, Δ144/145, Δ146, Δ210, and Δ243–244) and the unmodified protein (wild type), whereas 4A8 mAb (green) does not detect mutants containing deletions in RDR2 or RDR4 (Δ69–70+Δ141–144, Δ141–144, Δ144/145, Δ146, and Δ243–244). Overlay images (RBD/4A8/DAPI) depict colocalization of the antibodies; nuclei were counterstained with 4′,6-diamidino-2-phenylindole (DAPI; blue). Scale bars, 100 μm. (**C**) Virus isolated from PLTI1 resists neutralization by 4A8. A nondeletion variant (Munich) is neutralized by 4A8, both are neutralized by convalescent serum, and neither is neutralized by H2214, an influenza hemagglutinin binding antibody (*29*).

The recurrence and convergence of RDR deletions, particularly during long-term infections, is indicative of adaptation in response to a common selective pressure. RDRs 2 and 4 and RDRs 1 and 3 occupy two distinct surfaces on the S glycoprotein NTD (Fig. 4A). Both sites contain antibody epitopes (*17*–*19*). The epitope for neutralizing antibody 4A8 is formed entirely by the β sheets and extended connecting loops that harbor RDRs 2 and 4 (*17*). We generated a panel of S glycoprotein mutants representing the four RDRs to assess the impact of deletions on expression and antibody binding; we included an additional double mutant containing the deletions present in the B.1.1.7 variant of concern flagged initially in the United Kingdom. Cells were transfected with plasmids expressing these mutant glycoproteins, and indirect immunofluorescence was used to determine whether RDR deletions modulated 4A8 binding (Fig. 4B). Deletions at RDRs 1 and 3 had no impact on the binding of the monoclonal antibody, confirming that they alter independent sites. The three RDR2 deletions, the one RDR4 deletion, and the double RDR1/2 deletions completely abolished binding of 4A8 while still allowing recognition by a monoclonal antibody targeting the RBD (Fig. 4B). Thus, convergent evolution operates in individual RDRs and between RDRs, as exemplified by the same phenotype produced by deletions in RDR2 or RDR4.

We used the non–plaque-purified viral population from PLTI1 to determine whether RDR variants escape the activity of a neutralizing antibody. This viral stock was completely resistant to neutralization by 4A8, whereas an isolate with authentic RDRs (*20*) was neutralized (Fig. 4C). We used a high-titer neutralizing human convalescent polyclonal antiserum to demonstrate that both viral stocks could be neutralized efficiently. These data demonstrate that naturally arising and circulating variants of SARS-CoV-2 have altered antigenicity. We used a range of high-, medium-, and low-titer neutralizing human convalescent polyclonal antisera to assess whether there was an appreciable difference in neutralization between the S glycoprotein–deleted and undeleted viruses. No major difference was observed, which suggests that many more changes would be required to generate serologically distinct SARS-CoV-2 variants (table S1).

Coronaviruses, including SARS-CoV-2, have lower substitution rates than other RNA viruses because of an RdRp with proofreading activity (*10*, *11*). However, proofreading cannot correct deletions. We find that adaptive evolution of S glycoprotein is augmented by a tolerance for deletions, particularly within RDRs. The RDRs occupy defined antibody epitopes within the NTD (*17*–*19*), and deletions at multiple sites confer resistance to a neutralizing antibody. Deletions represent a generalizable mechanism through which S glycoprotein rapidly acquires genetic and antigenic novelty of SARS-CoV-2.

The fitness of RDR variants is evident by their representation in the consensus genomes from patients, transmission between individuals, and presence in emergent lineages. Initially documented in the context of long-term infections of immunosuppressed patients, specific variants transmit efficiently between immunocompetent individuals. Characterization of these cases led to the very early identification of RDR variants that are escape mutants. Because deletions are a product of replication, they will occur at a certain rate and variants are likely to emerge in otherwise healthy populations. Indeed, influenza explores variation that approximates future antigenic drift in immunosuppressed patients (*21*).

The RDRs occupy defined antibody epitopes within the S glycoprotein NTD. Selected in vivo, these deletion variants resist neutralization by monoclonal antibodies. Viruses cultured in vitro in the presence of immune serum have also acquired substitutions in RDR2 that confer neutralization resistance (*22*). Potent neutralizing responses and an array of monoclonal antibodies are directed to the RBD (*18*, *19*, *23*). A growing number of NTD-directed antibodies have been identified (*24*, *25*). Why antibody escape in nature is most evident in the NTD highlights a discrepancy, and this requires further study.

Defining recurrent, convergent patterns of adaptation can provide predictive potential. From viral sequences, we have identified a pattern of deletions, contextualized their outcomes in protein structure and antibody epitope(s), and characterized their functional impact on antigenicity. During evaluation of this manuscript, multiple lineages with altered antigenicity and perhaps increased transmissibility have emerged and spread. These variants of global concern are RDR variants and include Mink Cluster 5 Δ69–70 (*26*), B.1.1.7 Δ69–70, and Δ144/145 (*27*), as well as B.1.351 Δ242–244 (*28*). Our analysis preceded the description of these lineages. We had demonstrated that identical or similar recurrent deletions that alter positions 144/145 and 243–244 in the S glycoprotein disrupt binding of antibody 4A8, which defines an immunodominant epitope within the NTD. Our survey for deletion variants captured the first representative of what would become the B.1.1.7 lineage. These real-world outcomes demonstrate the predictive potential of this and like approaches and show the need to monitor viral evolution carefully and continually.

Additional circulating RDR variants have gone virtually unnoticed. Are they intermediates on a pathway of immune evasion? That remains to be determined. However, deletions and substitutions within major NTD and RBD epitopes will likely continue to contribute to that process, as they have already in current variants of concern. The progression of adaptations in both immunocompromised patients and SARS-CoV-2 variants of concern remains to be resolved. Their evolution has thus far converged. The recurrence of adaptations in single patients and on global scales underscores the need to track and monitor deletion variants.

## Supplementary Materials

science.sciencemag.org/content/371/6534/1139/suppl/DC1 Materials and Methods Figs. S1 to S3 Tables S1 and S2 References (*30*–*34*) MDAR Reproducibility Checklist
